# Characterization of the bacterial microbiome of Swedish ticks through 16S rRNA amplicon sequencing of whole ticks and of individual tick organs

**DOI:** 10.1186/s13071-022-05638-4

**Published:** 2023-01-30

**Authors:** Giulio Grandi, Giulia Chiappa, Karin Ullman, Per-Eric Lindgren, Emanuela Olivieri, Davide Sassera, Emma Östlund, Anna Omazic, Debora Perissinotto, Robert Söderlund

**Affiliations:** 1 Department of Microbiology, National Veterinary Institute (SVA), 751 89 Uppsala, Sweden; 2 Department of Biomedical Sciences and Veterinary Public Health (BVF), Swedish University of Agricultural Sciences (SLU), Ulls Väg 26, 750 07 Uppsala, Sweden; 3 Department of Biomedical and Clinical Sciences, Division of Inflammation and Infection, Linköping University, 581 85 Linköping, Sweden; 4 Department of Clinical Microbiology, County Hospital Ryhov, 551 85 Jönköping, Sweden; 5 Istituto Zooprofilattico Sperimentale della Lombardia e dell’Emilia Romagna, Strada Campeggi, 59/61, 27100 Pavia, Italy; 6 Department of Biology and Biotechnology “L. Spallanzani”, University of Pavia, Pavia, Italy; 7 Department of Chemistry, Environment, and Feed Hygiene, National Veterinary Institute (SVA), 751 89 Uppsala, Sweden

**Keywords:** *Ixodes ricinus*, Microbiota, Community profiling, Tick-borne pathogens, One Health, NGS, 16S, Endosymbiont, *Borrelia*, Midichloria

## Abstract

**Background:**

The composition of the microbial flora associated with ixodid ticks has been studied in several species, revealing the importance of geographical origin, developmental stage(s) and feeding status of the tick, as well as substantial differences between tissues and organs. Studying the microbiome in the correct context and scale is therefore necessary for understanding the interactions between tick-borne pathogens and other microorganisms as well as other aspects of tick biology.

**Methods:**

In the present study the microbial flora of whole *Ixodes ricinus*, *I. persulcatus* and *I. trianguliceps* ticks were analyzed with 16S rRNA amplicon sequencing. Additionally, tick organs (midguts, Malpighian tubules, ovaries, salivary glands) from flat and engorged *I. ricinus* female ticks were examined with the same methodology.

**Results:**

The most abundant bacteria belonged to the group of Proteobacteria (*Cand.* Midichloria mitochondrii and *Cand.* Lariskella). 16S amplicon sequencing of dissected tick organs provided more information on the diversity of *I. ricinus*-associated microbial flora, especially when organs were collected from engorged ticks. Bacterial genera significantly associated with tick feeding status as well as genera associated with the presence of tick-borne pathogens were identified.

**Conclusions:**

These results contribute to the knowledge of microbial flora associated with ixodid ticks in their northernmost distribution limit in Europe and opens new perspectives for other investigations on the function of these bacteria, including those using other approaches like in vitro cultivation and in vitro models.

**Graphical Abstract:**

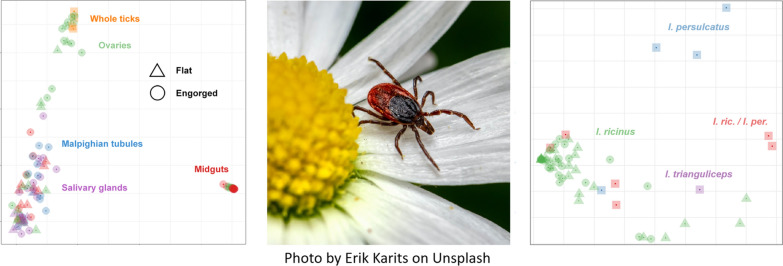

**Supplementary Information:**

The online version contains supplementary material available at 10.1186/s13071-022-05638-4.

## Introduction

Ticks (Acari: Ixodida) are vectors for an array of tick-borne pathogens (TBPs) able to cause several tick-borne diseases (TBD) in humans and animals worldwide. Hard ticks (Ixodidae) are well recognized as vectors for viruses, bacteria, protozoa and helminths that can cause different pathological outcomes well described in humans (i.e. Lyme borreliosis, tick-borne encephalitis and granulocytic anaplasmosis), in domesticated animals (i.e. granulocytic anaplasmosis, babesiosis) and to a lesser extent in wildlife [1]. Beside TBPs, ticks harbour other microorganisms—commensals and endosymbionts, the latter vertically inherited, with the whole microbial flora associated with the tick referred to as the ‘tick microbiome’ [2, 3]. The functions of these bacteria are still largely unknown, but recent studies are confirming the hypothesis that they most likely support tick fitness by providing nutrients that hematophagous arthropods like ticks are not able to synthesize, such as vitamins of the B group or other compounds [4, 5]. The tick microbiota also appears to play a role in the establishment of infection and/or transmission of TBPs [3], most likely through interaction with the tick’s immune system [6], and for this reason it has been hypothesized that control of tick populations or their TBPs could be achieved through manipulation of the microbiota in general and endosymbionts in particular [7, 8].

The complexity of tick microbiomes as systems has been studied only recently at the molecular level, with the majority of studies still tending to focus on the occurrence of a single TBP, only on some occasions studying co-infection between them [9] and often based on PCR detection techniques.

Recent studies on the bacterial flora associated with *I. ricinus* have been performed using metagenomics [14, 15], whole transcriptomics [16] or metatranscriptomics [17], using different developmental stages [18, 19]. In some cases, metagenomic studies were performed on *I. ricinus* dissected organs and different feeding status [20]. The most abundant bacteria in all these studies were the *Rickettsiales* (*Candidatus* Midichloria mitochondrii) followed by other bacteria belonging to this order of Proteobacteria [19]. The microbiome of *I. persulcatus* has also been investigated in several studies, and in this case the dominant bacterial symbiont found was again a *Rickettsiales, Candidatus* Lariskella arthropodarum [21, 22]. However, many questions remain regarding the complexity of the internal microbiome of hard ticks and the effect of feeding on the diversity and composition of bacteria in the midgut and other organs [6, 20, 23, 24], as well as the influence of technical challenges when performing community profiling of samples with limited bacterial abundance [23].

The present study was aimed at characterizing the bacterial microbiome in Swedish ticks and identifying differences associated with tick species, host species, feeding status and the presence of TBPs by using two different batches of samples: (i) whole ticks from northern Sweden (above latitude 60°N) collected through a citizen study in 2018 and (ii) tick organs obtained through dissection of ticks collected from central-southern Sweden in 2019–2020. Data on tick bacterial microbiome in Sweden are scarce and no previous studies have been done on material collected from such a large area of the country. Moreover, northern Sweden is a geographical region of particular interest since it is the northern distribution limit of *I. ricinus* and has recently been colonized by a new species, *I. persulcatus* [25]. Data obtained from different organs were gathered to facilitate future studies more focused on the functional aspects of tick microbiome.

## Methods

### Sample collection

#### Samples for whole tick analysis

More than 2000 ixodid ticks were collected between June and October in 2018 from northern Sweden (above latitude 60°N) to be screened for several TBPs. The ticks were collected by the public who had found them either on themselves or on domestic animals and in a few cases on wild animals. The ticks collected from the public were sent by mail to the National Veterinary Institute (SVA, Uppsala, Sweden), where they were stored separately at − 80 °C pending morphological identification according to literature [26, 27]. Identification of *I. ricinus* and *I. persulcatus* ticks was confirmed by PCR according to Michelet et al. [28]. A subsample of the ticks from this collection (*n* = 96) was selected for 16S sequencing: (i) all the *I. persulcatus* specimens (*n* = 20, *n* = 4 from cat and *n* = 16 from dogs), (ii) all ticks PCR positive for both *I. ricinus* and *I. persulcatus* (*n* = 7, *n* = 3 from cats and *n* = 4 from dogs), (iii) all the *I. trianguliceps* (*n* = 5: *n* = 2 from mice, *n* = 2 from vole and *n* = 1 from cat) and (iv) a selection of *I. ricinus* ticks from cats (*n* = 32) and dogs (*n* = 32). To have a sample representative of the geographical area of the collection, *I. ricinus* (the dominant species) specimens were selected from different localities. A list of the samples for the whole tick analysis, including tick species, developmental stage, sex, municipality of collection and host species, is provided as Additional file 1: Table S1.

#### Ticks for organ dissection

During the summer of 2020, female *I. ricinus* ticks (*n* = 36) were collected and kept alive to dissect them and analyze the collected organs with the same method used with the whole ticks (see “Sequencing and community profiling”). Ticks were collected from dogs and cats from the following sites: Uppsala (*n* = 14), Forsmark, Uppsala County (*n* = 7), Kyrkby, Uppsala County (*n* = 5), Stockholm (*n* = 3) and Tyringe, Skåne county (*n* = 7). A list of the samples for 16S analysis of tick organs, including feeding status, the organs collected from each specimen and the municipality/site of collection, is provided as Additional file 1: Table S1. Details on the host species were not available since the owners had both dogs and cats and rarely provided this information.

### Sample preparation

#### Homogenization and nucleic acid extraction of whole ticks

Before morphological identification, the ticks were individually washed with 70% ethanol solution followed by MilliQ water. Each tick was then incubated in 450 µl RLT-buffer (Qiagen, Hilden, Germany) supplemented with 40 mM dithiothreitol (DTT) together with a sterile 5-mm stainless-steel bead (Qiagen, Hilden, Germany) and homogenised in a Tissue Lyser (Qiagen, Hilden, Germany) for 2 min at 30 Hz. To extract total nucleic acids (NA), the homogenates were centrifuged for 3 min at 20,000×*g* and then 90 µl of the supernatant was mixed with 10 µl Proteinase K (Sigma-Aldrich, Germany). The extraction was performed in the Magnatrix 8000 + extraction robot (Magnetic BioSolutions, Stockholm, Sweden) with either of two commercial extraction kits: Bullet Stool kit 1.32.104 (Diasorin, Italy) or Vet NA kit 1.001 (Bioservices, Sweden).

#### Tick dissection and DNA extraction for tick organ analysis

Prior to processing, all ticks collected were surface sterilized by washing in 1% bleach followed by three successive baths of DNA-free water to denature the DNA of external bacteria [29]. Dissection of ticks was carried out as previously described [30]. Briefly, after removing the dorsal cuticle, specific organs including Malpighian tubules, midgut, ovaries and salivary glands were removed and placed in droplets of sterile phosphate-buffered saline (PBS) 1 × to wash them. Organs were then stored in 70% ethanol.

DNA extraction was performed from dissected organs using the NucleoSpin^®^ Kit (Macherey Nagel, Duren, Germany) following the manufacturer’s instructions.

### Sequencing and community profiling

16S ribosomal RNA community profiling was performed with libraries prepared according to the Illumina standard protocol [31] and sequenced with 300-bp paired-end V3 chemistry on a MiSeq instrument. Negative controls, i.e. blank sample library preparations, were included in each run. Microbiome analysis on the resulting data was performed using QIIME 2.0 [32] on the Nephele platform [33] with default settings. Biological observation matrix (biom) files from Nephele were processed with the Phyloseq package [34] in R 4.0.4 (r-project.org). Alpha diversity was calculated using the Chao1 and Shannon indices. For further analysis, any operational taxonomic unit (OTU) that constituted 0.1% or more of the observed OTUs in one or more negative control samples was considered to be a likely reagent or consumable contaminant and excluded from all further analyses. Samples with < 5000 read pairs after removal of likely contamination were excluded from further analysis. To further reduce spurious calls, e.g. from index hopping, any OTU with a count of less than five in a given sample was considered zero for that sample. Alpha diversity was calculated using the Chao1 and Shannon indices. Samples were compared by principal coordinate analysis (PCoA) using log-transformed OTU counts and the Bray-Curtis distance metric. PCoA results were visualized using the cowplot package. Heatmaps were generated showing the distribution of the most abundant OTUs aggregated to genus level between samples. Normalised OTU data aggregated to order level were used to produce barplots; OTUs which could not be classified to this level were excluded from the plots. Significance testing was performed to identify OTUs associated with the gut of engorged ticks vs. flat ticks and to identify OTUs significantly more or less abundant in samples positive for TBPs using the DESeq2 package [35] with the Wald test. For the latter case, a correction for systematic differences between sample types was made by including this factor as an explanatory variable in the DESeq2 analysis. Only OTUs with a base mean of > 500 were included in the significance testing for engorged vs. flat ticks. A cutoff of *P* = 0.01 was used with *P*-values adjusted with the Benjamini-Hochberg method [36] to account for testing of multiple hypotheses.

## Results

### 16S community profiling of whole ticks

**Fig. 1:**
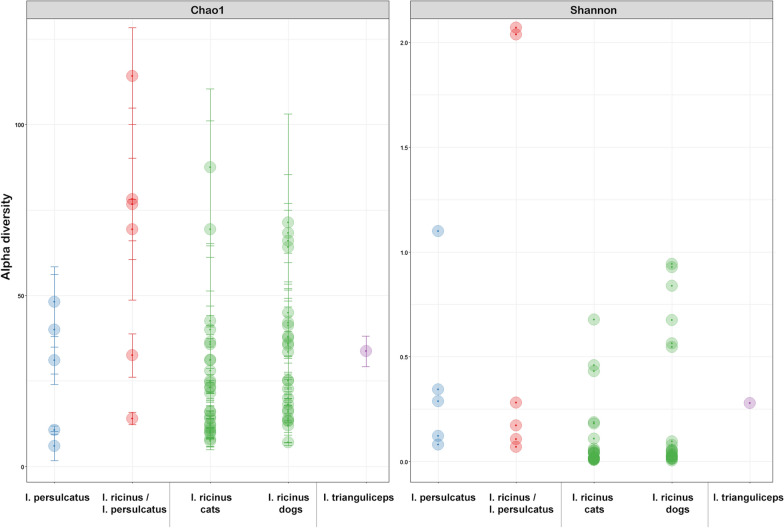
Alpha diversity for 16S rRNA OTU data from whole ticks, calculated as the Chao1 (left) and Shannon (right) measures using phyloseq in R

**Fig. 2:**
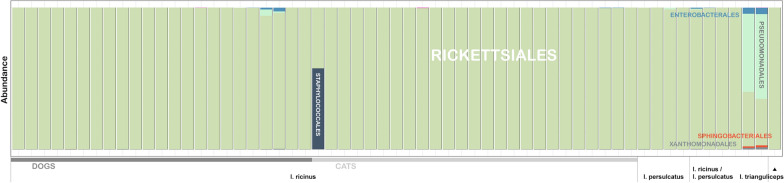
Barplot showing relative abundance of OTUs aggregated to the taxonomic level of order in samples and sample categories of whole ticks. Most samples are dominated by *Rickettsiales*, primarily representing endosymbionts. Bars do not sum to 100% as certain OTUs could not be identified to order level

**Fig. 3:**
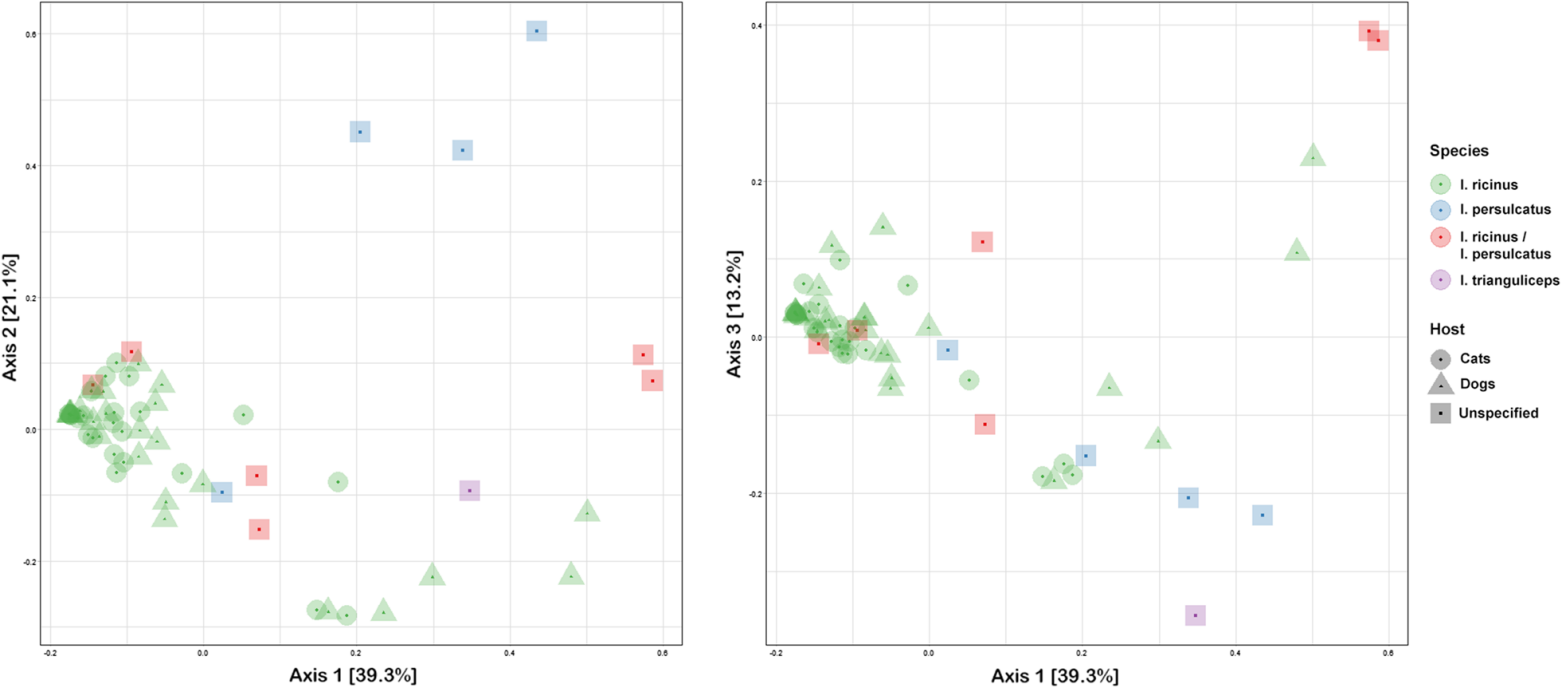
Principal coordinate analysis of 16S rRNA OTU data from whole ticks, showing the first three axes (left: axis 1 vs. axis 2, right: axis 1 vs. axis 3). A degree of clustering by tick species is evident, likely due to the presence and abundance of endosymbiont species, but no effect of feeding host (cat vs. dog)

Overall community diversity was low in 16S rRNA sequenced from 59 whole ticks from which sufficient read counts could be extracted (Fig. 1; Additional file 1: Table S1), with most samples dominated by likely endosymbiont species (Fig. 2; Additional file 3: Fig. S1). *Midichloria* occurred in all ticks except a single *I. persulcatus* and was especially prevalent in *I. ricinus* ticks. *Lariskella* was common in most *I. persulcatus*. Known or putative tick-borne pathogens (TBPs) were detected in several samples: *Anaplasma* (*n* = 6), *Borrelia* (referred to as *Borreliella* in the sequence library) (*n* = 2) and *Neoehrlichia* (*n* = 2) (Additional file 1: Table S1). Notably, the majority of 16S sequences observed in the single *I. trianguliceps* tick were from *Anaplasma* (Fig. 2). Additionally, there were several *Rickettsia* spp*.* that could not be determined to species level. *Pseudomonas* occurred in all tick species. Other recurring findings were *Enterobacterales*, *Sphingobacterium* and *Stenotrophomonas*. For the most part microbiomes clustered by tick species when analysed with PCoA (Fig. 3), but there was no evident separation of *I. ricinus* ticks from cats and dogs. Of the ticks with molecular markers consistent with both *I. ricinus* and *I. persulcatus*, four produced mainly *Midichloria* reads and clustered with the *I. ricinus* samples, while two clustered separately and were dominated by *Pseudomonas*, *Midichloria* and *Lariskella* (Figs. 2, 3). Due to the low diversity statistical analysis was not deemed meaningful for the 16S data from whole ticks.

### 16S community profiling of tick organs

**Fig. 4:**
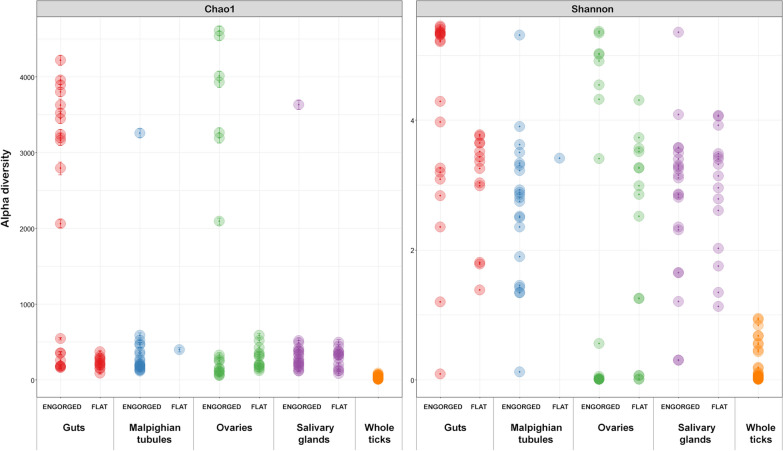
Alpha diversity for 16S rRNA OTU data from tick organs collected from engorged and flat ticks, calculated as the Chao1 (left) and Shannon (right) measures using phyloseq in R. Data from a set of whole ticks from the previous study (Fig. 1) are included for comparison. Diversity is higher in all organ categories compared to whole ticks and particularly high in certain organs from engorged ticks

**Fig. 5:**
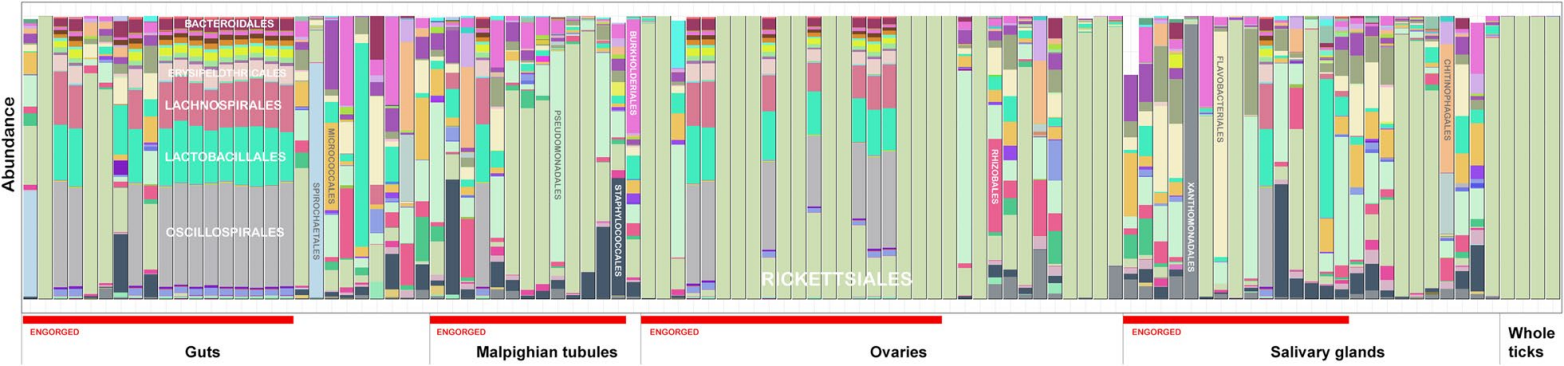
Barplot showing relative abundance of OTUs aggregated to the taxonomic level of order in samples and sample categories of tick organs. Data from a set of whole ticks from the previous study (Fig. 2) are included for comparison. Bars do not sum to 100% as certain OTUs could not be identified to order level

**Fig. 6:**
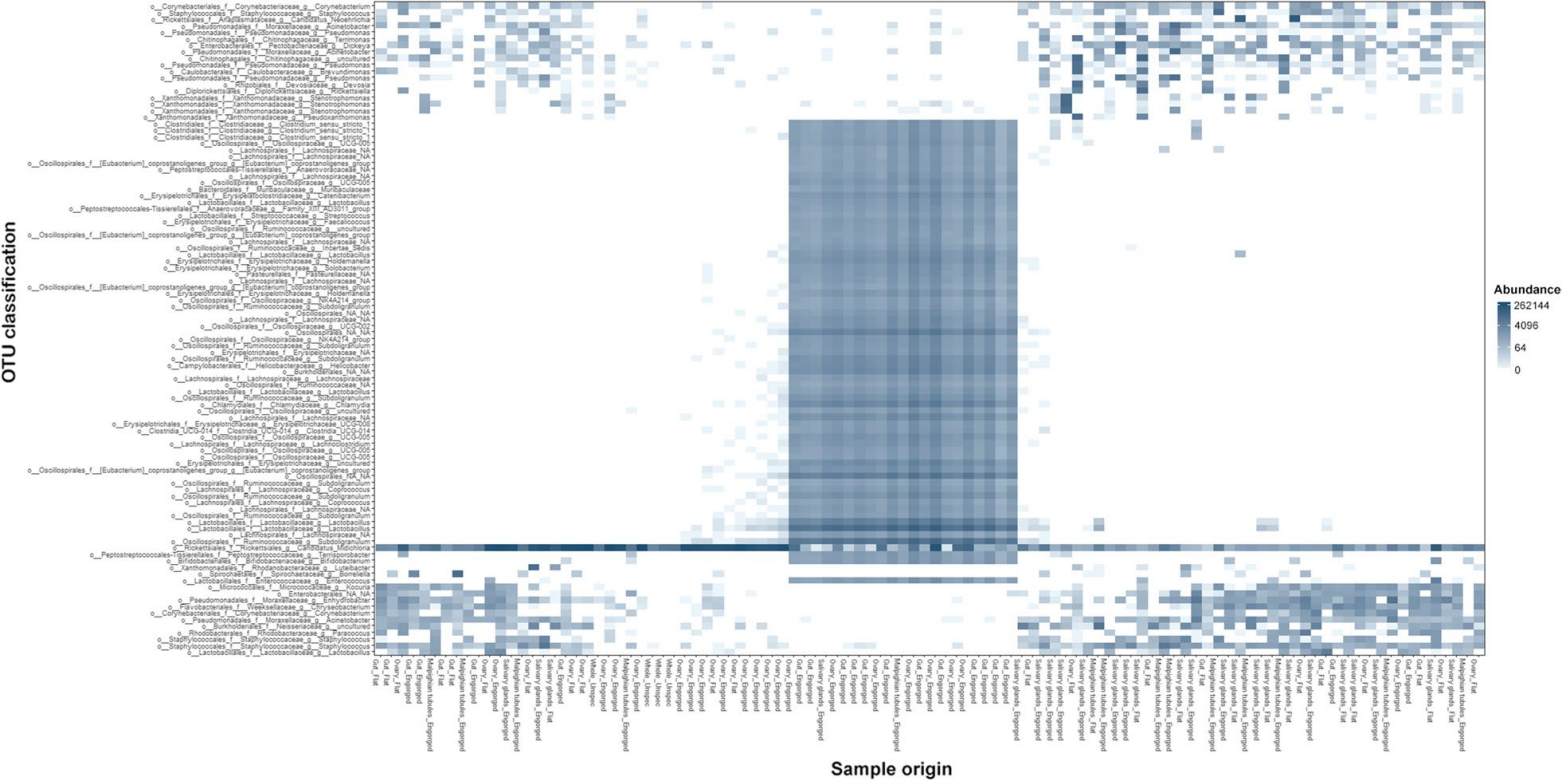
Heatmap of the abundance per sample of the 500 most abundant OTUs overall in data from whole *I. ricinus* ticks, clustering samples by abundance profile. A cluster of organ samples, all from engorged ticks, shows a high abundance of a shared set of several OTUs. The horizontal line in the lower part of the heatmap corresponds to *Midichloria*

**Fig. 7:**
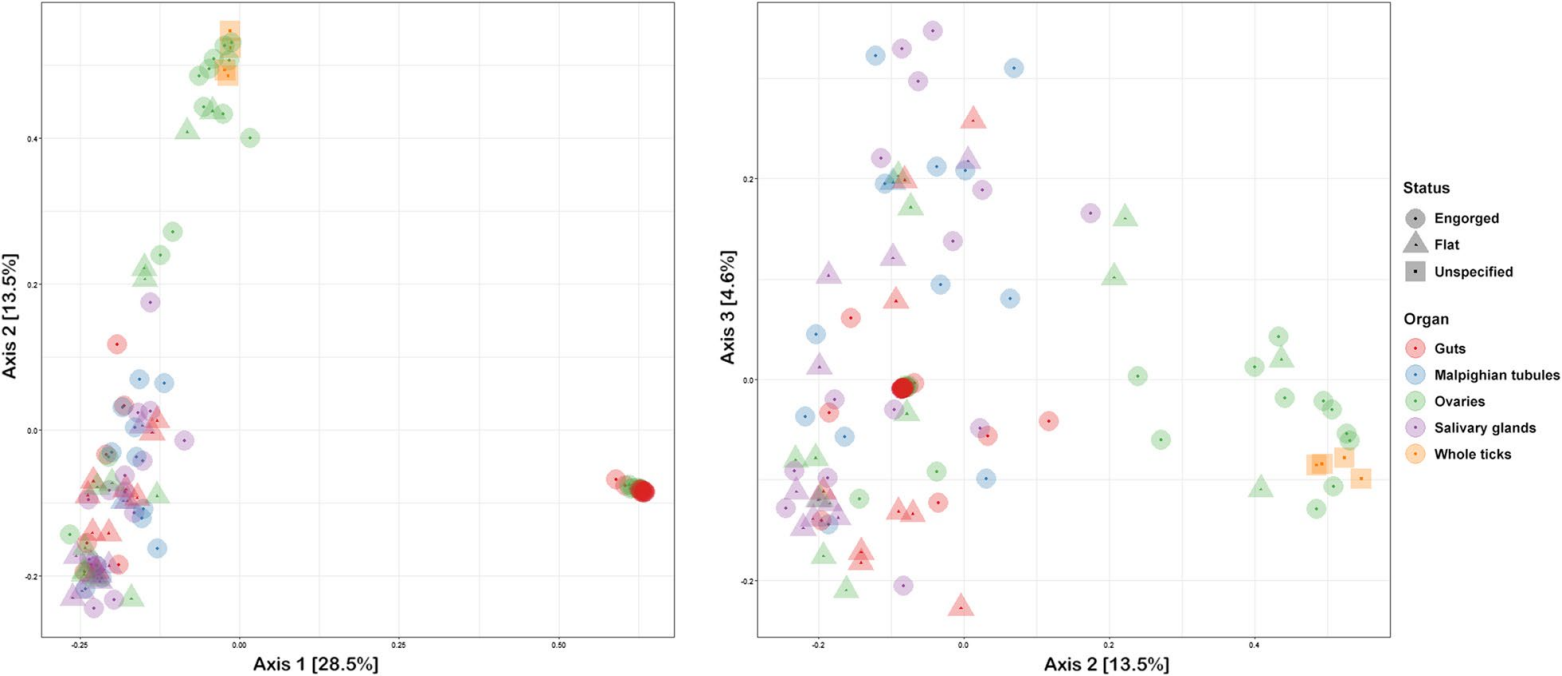
Principal coordinate analysis of 16S rRNA OTU data from tick organs, showing the first three axes (left: axis 1 vs. axis 2, right: axis 2 vs. axis 3). Data from a set of whole ticks from the previous study (Fig. 3) are included for comparison. Clustering by both organ category and especially feeding status is evident

Sufficient read counts for community profiling were produced from a total of 98 organ samples (Additional file 1: Table S1). The microbiota of all categories of organ samples were more diverse than those of samples consisting of whole ticks, with the highest diversity observed in samples from the guts of engorged ticks (Figs. 4, 5), although differing numbers of reads between experiments and sample categories likely influence diversity metrics to some extent complicating direct comparison. Most samples regardless of organ type contained a significant number of *Midichloria* 16S sequences, but in contrast with the whole ticks this genus was only the most abundant in a set of samples primarily from ovaries but also from salivary glands (Fig. 5). Seven samples were positive for *Ricketsiella* in addition to *Midichloria*, while *Lariskella* was absent from all samples. The most common observations overall included *Enhydrobacter*, *Acinetobacter*, *Stenotrophomonas*, *Streptococcus*, *Pseudomonas, Brevundimonas, Dickeya, Chryseobacterium* and bacteria of the *Chitinophagaceae* and *Neisseriaceae* families (Additional file 4: Fig. S2). Most samples from the guts of engorged ticks as well as a number of samples from other organs collected from engorged ticks presented a remarkably stable composition of OTUs both in terms of presence and relative abundance (Fig. 5; Additional file 4: Fig. S2). This conserved bacterial community was dominated by *Lachnospirales*, *Oscillospirales*, *Bacteroidales*, *Lactobacillales*, *Christensenellales*, *Peptostreptococcales-Tissierellales* and *Clostridiales,* but contained representatives of many other orders (Fig. 6; Additional file 4: Fig. S2). There was also a trend of more reads being generated from engorged tick guts, possibly associated with a higher number of bacteria overall in these samples (Additional file 5: Fig. S3). DESeq2 testing identified 2093 OTUs as significantly different in abundance between gut samples from flat and engorged ticks (*P* < 0.01, BH adjusted, Additional file 2: Table S2). Consistent with this, PCoA produced a tight cluster consisting of gut and other organ samples from engorged ticks (Fig. 7). Several ovary samples clustered closest to the reference samples from whole ticks were included for comparison, while no clear separation was evident for the other sample types.

Putative or known TBPs were detected by 16S sequencing in 25 organ samples (Additional file 1: Table S1). Of these, nine samples were positive for *Borrelia* and 14 for *Neoehrlichia*, one of which was positive for both. *Borrelia* was most commonly detected in ovaries (*n* = 5) but also salivary glands (*n* = 3) and one gut sample. A single tick was positive in both the salivary glands and ovaries. *Neoehrlichia* was detected in all sample types: gut samples (*n* = 4), ovaries (*n* = 4), salivary glands (*n* = 3) and Malpighian tubules (*n* = 3). Two ticks were positive for *Neoehrlichia* in two organs: salivary glands together with gut or ovary. Two samples were positive for *Rickettsia* and two for *Diplorickettsia,* one of which was also positive for *Neoehrlichia*. *Anaplasma* was not detected in any organ sample. A total of 46 OTUs were significantly differentially abundant in samples positive for *Borrelia* (*P* < 0.01, BH adjusted, Additional file 2: Table S2), with the strongest positive correlation observed for representatives of *Pedobacter*, *Neisseriaceae* and *Aerococcaceae* and the strongest negative associations with *Rhizobium*, *Paracoccus* and *Pseudomonas*. Thirty significant OTUs were identified for *Neoehrlichia*, with positive associations for *Pseudoxanthomonas*, *Devosia* and *Pseudomonas* and negative for *Comamonadaceae*, *Granulicatella* and *Roseomonas* (Additional file 2: Table S2). Statistical analysis was not performed for the other TBPs because of the low number of positive samples.

## Discussion

In recent years, technological advances combined with an increasing understanding of the influence of the host microbiome on carriage and transmission of vector-borne pathogens have contributed to a rising interest in studying the bacterial flora of ticks. The tick microbiome must however be considered in the appropriate context and scale for a meaningful biological interpretation to be possible (reviewed by [3, 37]). Apart from the geographical origin, life stage and sex of the tick, the choice of tissue studied also has a major impact on the results [38]. Due to the difficulties associated with dissection and genetic characterization of small sample quantities, most studies of tick microbiomes have been performed on whole ticks [14, 16, 18, 19, 21, 22]. In the present study, we initially performed 16S rRNA community profiling of whole ticks of the species *I. ricinus*, as well as a smaller number of *I. persulcatus* and *trianguliceps* and ticks with ambiguous species identity. The generated data consisted to a large extent of observations of likely endosymbionts with *Midichloria* highly abundant in all *I. ricinus* samples, consistent with previous observations that these bacteria are present in virtually all female *I. ricinus* [39] as well as in several related tick species [40]. In contrast, the microbiomes of *I. persulcatus* ticks and ticks with molecular markers consistent with both *I. ricinus* and *I. persulcatus* were composed of varying proportions of *Midichloria* together with the related endosymbiont *Lariskella. Lariskella* is known to occur in *I. persulcatus* as well as in *I. ricinus*/*I. persulcatus* hybrid ticks [41]. Unidentified *Rickettsia* bacteria also occurred in multiple samples, which may represent further endosymbionts, potential TBPs or indeed both as the full spectrum is represented in this diverse genus of obligately intracellular bacteria [42]. Known TBPs were also detected in several samples. For *I. ricinus* whole ticks, data on the host animal species (dog or cat) were available but this was found not to influence the microbiota to a detectable degree. Possible effects on microbial community composition in ticks fed on different host species have been reported, but generally in cases of markedly different host animals or related to differences in feeding strategies (reviewed in [6]). Any such differences that did not involve the major endosymbiont species would have had to be strong to be visible in the present study.

Considering the high proportion of endosymbiont-derived reads in the whole tick data, a second 16S dataset was generated with samples consisting of organs from *I. ricinus* ticks. The microbial diversity was higher in all categories of organs compared to whole ticks, and *Midichloria* was less dominant. For certain samples, and particularly ovaries, *Midichloria* were still the most common bacteria observed. This could be a consequence of a limited quantity of bacteria overall in these samples, but also reflect differences in *Midichloria* density between tissue categories and individual ticks, consistent with previous observations from quantitative PCR analysis of variation in the *Midichloria* density over several orders of magnitudes between individual ticks and tissue categories, with the highest abundance observed in ovaries [43]. A general trend was the presence of bacteria known to commonly occur in the environment (e.g. *Pseudomonas*) to variable degrees across all sample types and conditions, a phenomenon that has been repeatedly observed in previous studies [2, 38]. It is uncertain to what extent these bacteria represent true internal microflora, contaminants or both; it is certainly not unreasonable to assume a continuous exchange of bacteria among the tick’s environment, external surfaces, internal organs and the host. Some caution is necessary when interpreting data from small sample quantities or samples with low number of bacteria, as the 16S sequencing workflow includes an amplification step that will enhance any contaminants in the absence of relevant microflora [23, 44]. In addition to contaminants in the samples themselves, techniques based on 16S amplification are also vulnerable to contaminating bacterial DNA, which is frequently present in the reagents used [44, 45], and to between-sample leakage of sequences due to “index hopping” [46], although the rigorous filtering steps used in the present study likely reduce these issues.

16S sequences consistent with TBPs were recovered from all organ types sampled at varying frequencies, and in some cases from multiple organs in the same tick. Cross-contamination between tissues, e.g. during dissection, could be a factor explaining unexpected findings, such as the presence of *Borrelia* in ovaries. The presence of pathogens was linked to significantly higher or lower abundance of certain OTUs. Many of these were known environmental bacteria, e.g. associated with soil, water or plants. This could be indicative of certain commensals creating an environment differentially favourable for TBP colonization as has been observed for *Borrelia* [42]. Alternatively, active alterations in the microbiome by the TBPs themselves have been suggested, e.g. for *Anaplasma* in the gut of *Ixodes scapularis* [47]. An interesting finding in the present study is the negative association between *Borrelia* and the alphaproteobacterial genus *Paracoccus*, which have been found in a broad range of environmental samples [48] but are also prevalent in Brazilian *Amblyomma cajennense* ticks and have been suggested to be potential TBPs or to serve a role in the biology of the ticks [49]. Due to the low number of tick organs positive for TBPs and the need to correct for variation between organ categories, these results should be verified in a larger sample material. In general, the interpretation of negative correlations between TBPs and potential environmental contaminants is complicated by the fact that higher counts of other bacteria will lower the sensitivity of TBP detection.

Perhaps the most striking result in the present study was the significant association between tick engorgement and a broad but distinct set of OTUs, especially notable in the gut but also to some extent in other organs. Major components of this post-feeding flora are consistent with known members of the gut microbial community in other animals, e.g. *Ruminococcaceae*, *Clostridiales*, *Lachnospiraceae*, *Coprococcus*, *Clostridiales*, *Sharpea*, *Olsenella* and *Prevotella* in sheep [50], the uncultured *Muribaculaceae* CAG-873 in mice [51] and *Faecalibacterium*, *Subdoligranulum* and *Collinsella* in the human gut [52–54]. Although the midgut microbiome of unfed hard ticks in the form of *I. scapularis* has been described as limited [23], the blood meal creates a temporary environment with a relatively predictable profile of nutrients. This could explain the high degree of similarity between the microbial composition of engorged gut samples. Our results are in contrast with previous findings of limited diversity and increasing dominance of endosymbionts in engorged whole ticks of other species [55], possibly a result of the signal from the gut being drowned out by the remaining tick and particularly potentially dramatic expansion of endosymbiont populations in certain tissues after feeding [43].

## Conclusions

The presented data confirm previous results obtained from the same tick species in other geographical areas and adds new data to a growing knowledge about the association among the tick microbiome, tick borne pathogens and tick hosts.

## Supplementary Information

**Additional file 1: Table S1.** Metadata for ticks and tick organ samples analysed in the study

**Additional file 2: Table S2.** OTUs significantly associated with the presence of TBPs (*Borrelia*, *Neoehrlichia*) and OTUs significantly associated with feeding status in midgut samples

**Additional file 3: Figure S1.** Heatmap of the abundance per sample of OTUs in data from whole ticks, clustering samples by abundance profile.

**Additional file 4: Figure S2.** Heatmap of the abundance per sample of the 100 most abundant OTUs overall in data from whole *I. ricinus* ticks, clustering samples by abundance profile. A cluster of organ samples, all from engorged ticks, shows a high abundance of a shared set of several OTUs.

**Additional file 5: Figure S3**. Stacked histograms showing the distribution of read counts after filtering per sample category for whole ticks (upper panel) and tick organ samples (lower panel). Note different scale on x axes.

## Data Availability

The sequence datasets generated and analysed during the current study are available in the European Nucleotide Archive (https://www.ebi.ac.uk/ena) under project accession number PRJEB57195. Further sample data and analysis results are presented in Additional file 1, 2: Tables S1, S2.
