# Supplementary figures and images for: Characterization of the bacterial microbiome of Swedish ticks through 16S rRNA amplicon sequencing of whole ticks and of individual tick organs
Source: Parasit Vectors. 2023 Jan 30;16:39. doi: 10.1186/s13071-022-05638-4 (PMC9885626; doi:10.1186/s13071-022-05638-4)

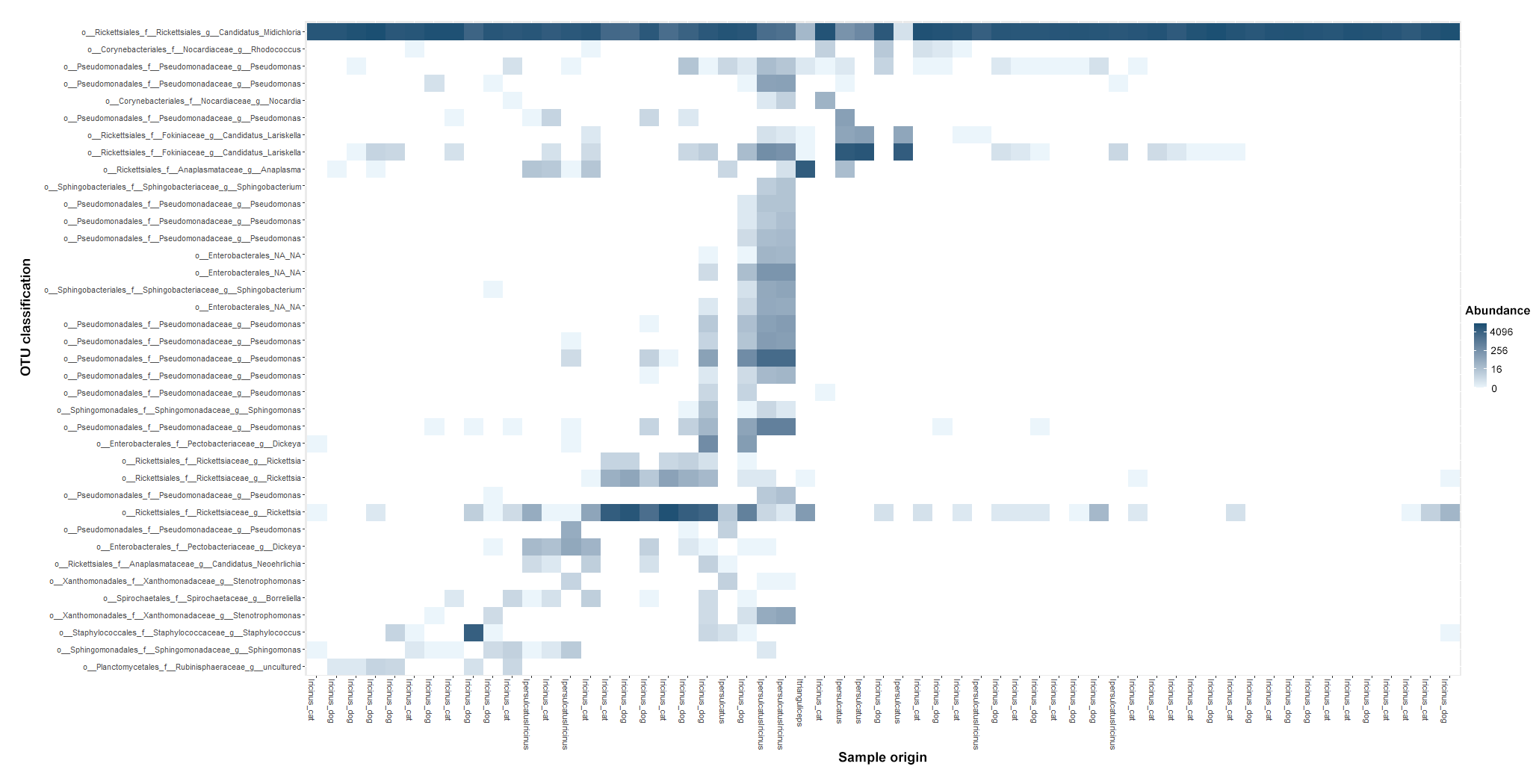

Supplement: Supplementary file 3 — Additional file 3: Figure S1. Heatmap of the abundance per sample of OTUs in data from whole ticks, clustering samples by abundance profile. [file 13071_2022_5638_MOESM3_ESM.png]

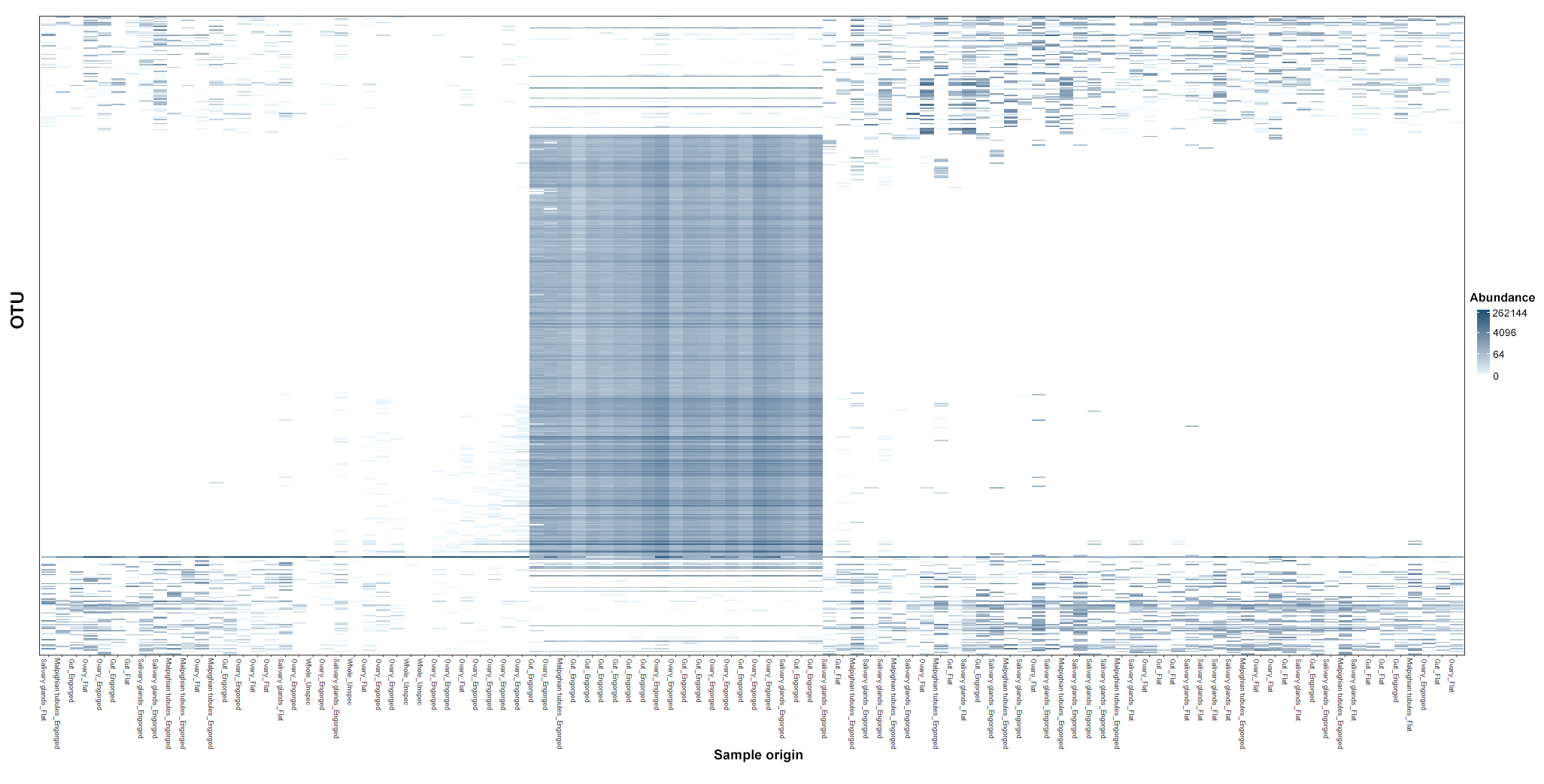

Supplement: Supplementary file 4 — Additional file 4: Figure S2. Heatmap of the abundance per sample of the 100 most abundant OTUs overall in data from whole I. ricinus ticks, clustering samples by abundance profile. A cluster of organ samples, all from engorged ticks, shows a high abundance of a shared set of several OTUs. [file 13071_2022_5638_MOESM4_ESM.png]

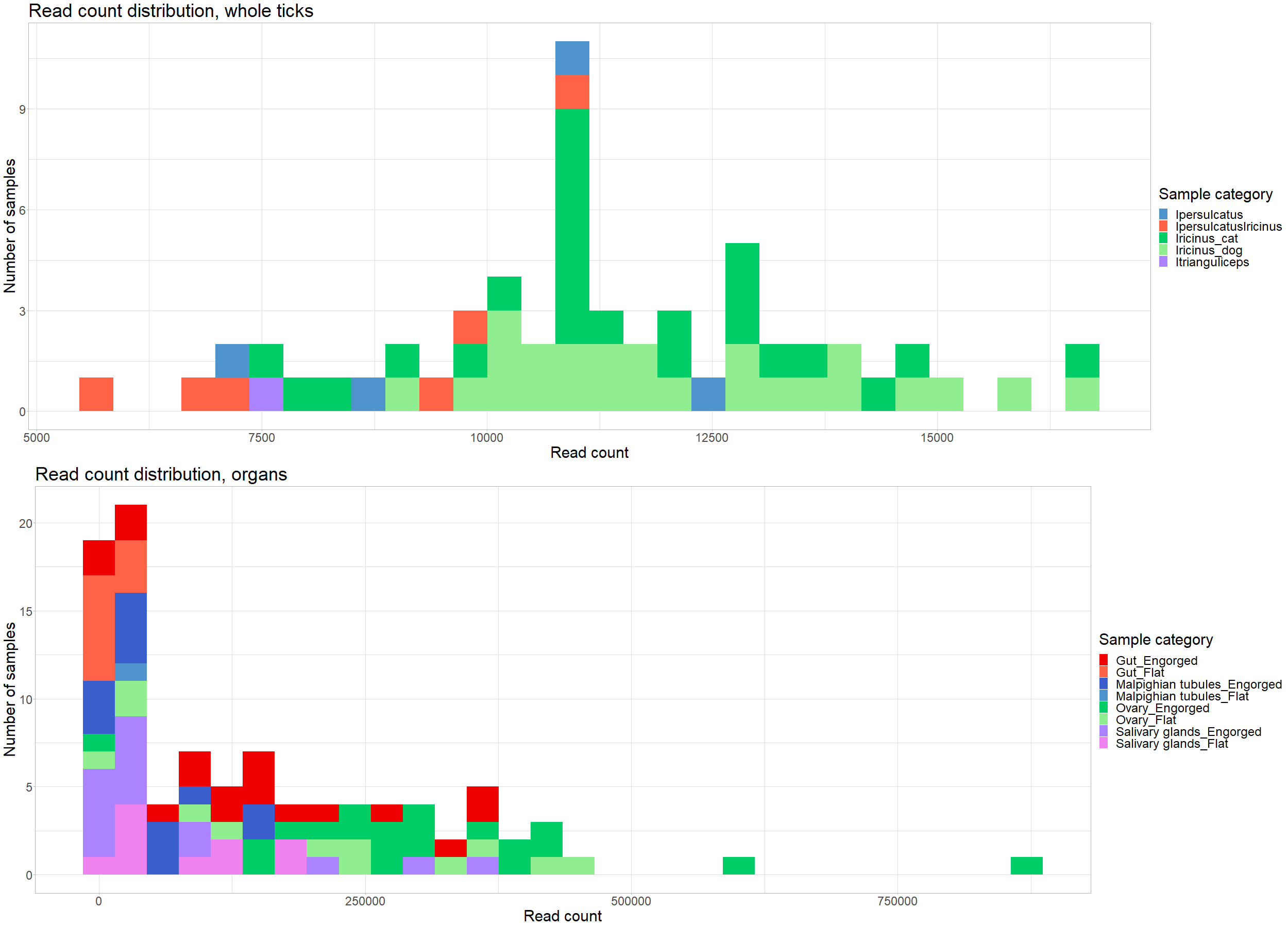

Supplement: Supplementary file 5 — Additional file 5: Figure S3. Stacked histograms showing the distribution of read counts after filtering per sample category for whole ticks (upper panel) and tick organ samples (lower panel). Note different scale on x axes. [file 13071_2022_5638_MOESM5_ESM.png]
